# Deoxyelephantopin Induces Apoptosis and Enhances Chemosensitivity of Colon Cancer via miR-205/Bcl2 Axis

**DOI:** 10.3390/ijms23095051

**Published:** 2022-05-02

**Authors:** Haoyan Ji, Kui Zhang, Guangzhao Pan, Changhong Li, Chongyang Li, Xin Hu, Liqun Yang, Hongjuan Cui

**Affiliations:** 1 State Key Laboratory of Silkworm Genome Biology, Medical Research Institute, Southwest University, Chongqing 400715, China; 18303478069@163.com (H.J.); zhangk87@163.com (K.Z.); m18983708534_2@163.com (G.P.); lichanghong960223@163.com (C.L.); chongyang1520@gmail.com (C.L.); huxinusing@163.com (X.H.); hcui@swu.edu.cn (H.C.); 2 College of Sericulture, Textile and Biomass Sciences, Southwest University, Chongqing 400716, China; 3 Cancer Center, Medical Research Institute, Southwest University, Chongqing 400716, China

**Keywords:** colon cancer, deoxyelephantopin, Bcl2, apoptosis, chemosensitivity

## Abstract

Colon cancer (CC) is one of the major causes of cancer death in humans. Despite recent advances in the management of CC, the prognosis is still poor and a new strategy for effective therapy is imperative. Deoxyelephantopin (DET), extracted from an important medicinal plant, *Elephantopus scaber* L., has been reported to exhibit excellent anti-inflammatory and -cancer activities, while the detailed anti-cancer mechanism remains unclear. Herein, we found that DET showed a significant CC inhibiting effect in vitro and in vivo without obvious organ toxicity. Mechanistically, DET inhibited CC cells and tumor growth by inducing G2/M phase arrest and subsequent apoptosis. DET-mediated cell cycle arrest was caused by severe DNA damage, and DET decreased the Bcl2 expression level in a dose-dependent manner to promote CC cell apoptosis, whereas restoring Bcl2 expression reduced apoptosis to a certain extent. Moreover, we identified a microRNA complementary to the 3′-UTR of Bcl2, miR-205, that responded to the DET treatment. An inhibitor of miR-205 could recover Bcl2 expression and promoted the survival of CC cells upon DET treatment. To further examine the potential value of the drug, we evaluated the combinative effects of DET and 5-Fluorouracil (5FU) through Jin’s formula and revealed that DET acted synergistically with 5FU, resulting in enhancing the chemotherapeutic sensitivity of CC to 5FU. Our results consolidate DET as a potent drug for the treatment of CC when it is used alone or combined with 5FU, and elucidate the importance of the miR-205-Bcl2 axis in DET treatment.

## 1. Introduction

Colon cancer (CC) is considered to be the third most prevalent cause of death from cancer after lung and breast cancers [1,2]. Although there has been a significant improvement in the treatment of colon cancer, the prognosis remains dismal. CC, as with many other solid tumors, progresses in stages and involves a variety of oncogenes and tumor suppressor genes [3]. Therefore, further investigation is required to understand the molecular mechanism of CC occurrence and develop novel effective therapeutic strategies.

Drug therapy is one of the main methods of tumor treatment. Many monomers of traditional medicine can inhibit migration and invasion and promote the apoptosis of tumor cells, which inhibits the occurrence and development of cancer. To date, increasing traditional medicine monomers have been used in clinical practice and the effect is remarkable. Therefore, drug therapy has good prospects for the treatment of cancer. However, current clinical treatments still have limitations, such as the serious side effects of chemotherapy drugs and tolerance to long-term use [4,5,6].

DET is a plant sesquiterpene lactone derived from Compositae *Elephantopus scaber* L. The first known report of DET in 1975 documented significant inhibitory activity on rat ascitic fluid [7]. Many subsequent reports highlighted the inhibitory effect of DET on a variety of cancer cells. For example, DET targets multimolecular signal transduction pathways to inhibit the growth and promote the apoptosis of cervical cancer SiHa cells [8]. DET also induces apoptosis of HCC cells through oxidative stress, inhibition of NF- kappa B, and mitochondrial dysfunction [9]. DET can inhibit the activity of triple-negative breast cancer cells through extracellular activity and protein function mediated by ROS [10]. Research has also witnessed an inhibitory effect of DET on the lung metastasis of mammary adenocarcinoma TS/A cells in mice [11]. DET is a sesquiterpene lactone, and the primary mechanism of almost all sesquiterpene lactones is the induction of oxidative stress for various biological and pharmacological activities [12,13,14,15,16,17,18,19,20]. Research shows that deoxyelephantopin induces ROS-mediated autophagy in human colorectal cancer in vitro and in vivo [21]. Herein, we report that DET presented an ideal colon cancer cell inhibition effect in vitro and in vivo without obvious organ toxicity. DET treatment inhibited the proliferation, colony formation, and cell cycle of colon cancer cells and repressed the progression of the tumor in the immune-deficient mouse model. Mechanistically, DET reduced the level of anti-apoptosis protein Bcl2, and the overexpression of Bcl2 inhibited apoptosis induced by DET. Further, we identified a microRNA complementary to the 3′-UTR of Bcl2, miR-205, which responded to the DET treatment and was upregulated. Finally, our results elucidate the importance of the miR-205-Bcl2 axis in DET treatment, and obtaining DET enhances the chemosensitivity of colon cancer to 5-Fluorouracil.

## 2. Results

### 2.1. DET Inhibits Colon Cancer without Obvious Organ Toxicity

**Figure 1:**
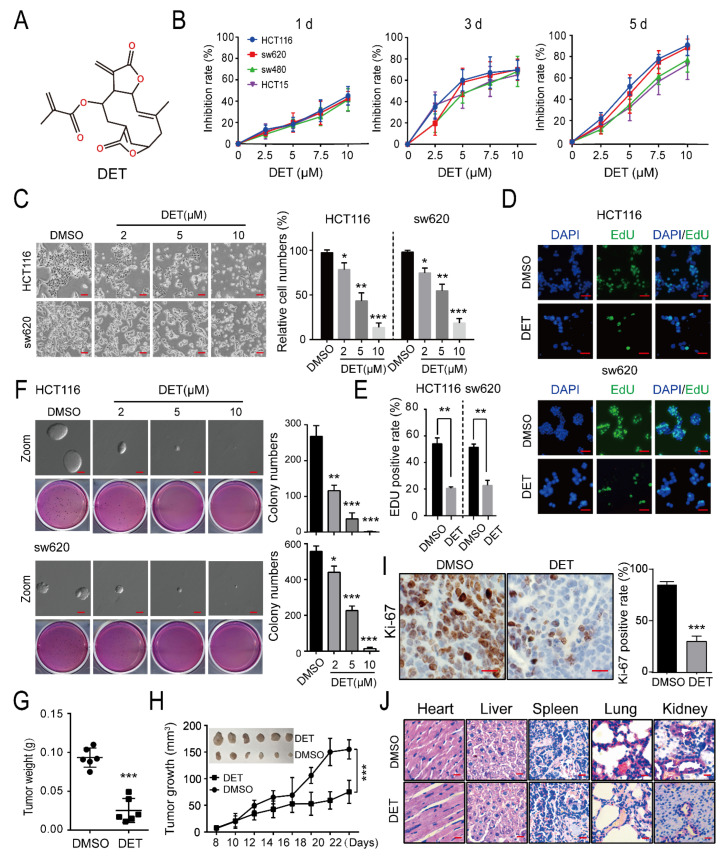
Deoxyelephantopin (DET) inhibits colon cancer without obvious organ toxicity. (**A**) The chemical structure of DET. (**B**) Graphic representation of results from MTT assays to determine cell inhibition rate of HCT116, sw620, sw480, and HCT15 cells. (**C**) Morphology of HCT116 and sw620 cells after treatment with DMSO or different concentrations of DET for 48 h. Scale bar = 20 µm. (**D**) Fluorescence images of EdU incorporation in HCT116 and sw620 cells treated with DET or DMSO for 48 h. Cells were stained to detect EdU (green) and DAPI (blue) to highlight nuclei, and images were superimposed. Scale bars = 20 µm. (**E**) The percentage of EdU+ cells (EdU+/DAPI+ × 100%) was evaluated in 4 random fields per sample. (**F**) The self-renewal capability of 5 µM DET-treated HCT116 and sw620 cells. Scale bar = 100 µm. (**G**) Measurement of the weight of the xenograft tumors. (**H**) The volume changes of xenograft tumors treated with DET. (**I**) The Ki-67 signal of mice was detected by immunohistochemistry. Scale bar = 20 µm. (**J**) H&E staining of the heart, liver, lung, spleen, and kidney in mice treated with DET or DMSO. Scale bar = 100 µm. All data are represented as the mean ±SD. A two-tailed unpaired Student’s *t*-test was carried out. * *p* < 0.05, ** *p* < 0.01, *** *p* < 0.001, versus control.

The chemical structural formula of DET is shown in Figure 1A. First, from the result of the 3-(4,5-Dimethylthiazol-2-yl)-2,5-diphenyltetrazolium bromide (MTT) assay shown in Figure 1B, it is apparent that there was a dose-dependent increase in the inhibitory effect of DET on the four types of colon cancer cells. Moreover, it was also evident that the inhibitory rate of DET on colon cancer cells had a time effect—namely, the inhibitory rate of cancer cells increased with the extension of time. Among the four cancer lines, the most prominent inhibitory rate changes were observed in HCT116 and sw620 cells. We treated HCT116 and sw620 with different concentrations of DET (2, 5, and 10 µM, and dimethyl sulfoxide (DMSO) was used as a control) for 48 h. Microscopic observation revealed a significant reduction in the number of alive cells of HCT116 and sw620 with the gradual increase in DET concentration (Figure 1C). Next, the results of the EdU assay, as shown in Figure 1D,E, suggested that the signals of cell proliferation in the DET group were lower than those in the control group, indicating the ability of DET to inhibit the DNA replication of HCT116 and sw620 cells.

Furthermore, the results of soft agar validated that the tumorigenic ability decreased apparently in a DET dose-dependent manner compared with the control group, which shows that DET can inhibit the self-renewal capability of colon cancer cells in vivo (Figure 1F). Meanwhile, we subcutaneously injected HCT116 cells into immunodeficient mice. A week later, the mice were randomly assigned to two groups: the control group and the DET treatment group. After two weeks, the xenograft tumors were removed and weighed. The results indicated that tumors in the DET group were significantly smaller as compared to those in the control group. The tumors’ weight and volume were also significantly lower than those in the control group (Figure 1G,H). Further, the immunohistochemistry (IHC) staining study showed that the Ki-67-positive signals that reflect the cell proliferation ability of the DET group were lower than those of the control group (Figure 1I). Together, these results confirmed that DET could inhibit the tumorigenesis of colon cancer cells in vitro and in vivo. Most importantly, as is evident in Figure 1J, mice organs did not manifest significant pathological alterations, indicating that DET had minimal side effects and little toxicity to mice’s visceral organs.

### 2.2. DET Arrests Colon Cancer Cells at the G2/M Phase by Inducing DNA Damage

**Figure 2:**
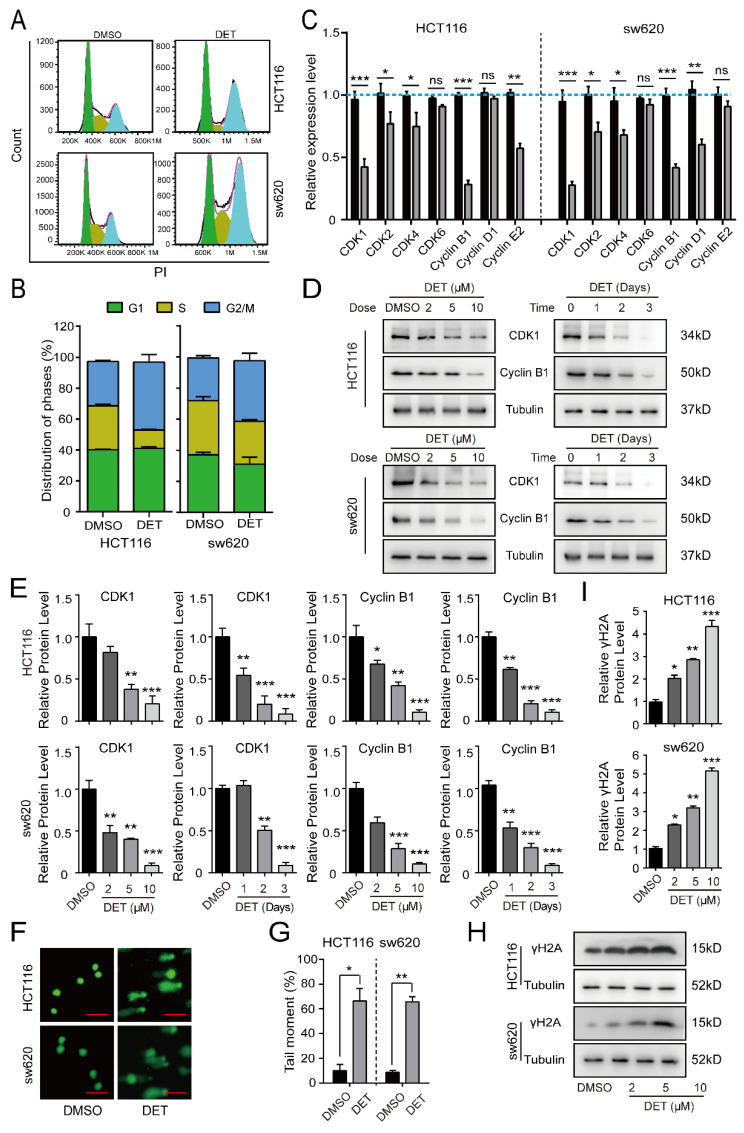
DET arrests colon cancer cells at the G2/M phase by inducing DNA damage. (**A**,**B**) The cell cycle of HCT116 and sw620 cells was analyzed by flow cytometry after treatment with DMSO or 5 µM of DET for 48 h. (**C**) Quantitative real-time PCR assay was conducted to identify the relevant genes regulating cell cycle after treatment of HCT116 and sw620 cells with DET. (**D**) Western blot assay was performed to evaluate the cell-cycle-related protein levels in the indicated concentrations (0, 2, 5, 10 µM) and indicated times (0, 24, 48, 72 h); GAPDH served as the control. (**E**) Protein levels were calculated based on the grayscale value of protein bands and normalized with the grayscale value of GAPDH bands. (**F**,**G**) Cell tailing exhibited by comet assay after DMSO or 5 µM of DET treatment for 48 h. Scale bar = 20 µm. (**H**,**I**) The expression level of γ H2A, a DNA-damage-specific marker, estimated by Western blot. All data are represented as mean ± SD. A two-tailed unpaired Student’s *t*-test was carried out. ns: not significant, * *p* < 0.05, ** *p* < 0.01, *** *p* < 0.001, versus control.

Since cell proliferation is tightly related to cell cycle progression, we explored the effect of DET on the cell cycle. As detailed in Figure 2A,B, cell cycles of HCT116 and sw620 cells treated with DET were arrested at the G2/M phase compared with the control group. Meanwhile, we detected the relevant genes regulating the cell cycle by qRT-PCR assay and identified the DET-induced significant downregulation of the mRNA level of CDK1 and CyclinB1, which regulate the G2/M phase (Figure 2C). Thereafter, detection of the cell cycle proteins by Western blot demonstrated the downregulation of protein levels of CDK1 and CyclinB1 in a dose-dependent and time-dependent manner (Figure 2D,E).

Further, DNA damage marks of DET-treated HCT116 and sw620 cells were evaluated due to the interference of the DNA damage response with cell cycle progression [22,23,24,25,26,27]. We observed that HCT116 and sw620 cells treated with DET demonstrated obvious tailing by the comet assay, which indicated DET-induced DNA damage in HCT116 and sw620 cells (Figure 2F,G). The Western blot assay also confirmed that the expression level of γ H2A, a DNA-damage-specific marker, was upregulated in a DET dose-dependent manner (Figure 2H,I).

### 2.3. DET Induces Apoptosis in Colon Cancer Cells

**Figure 3:**
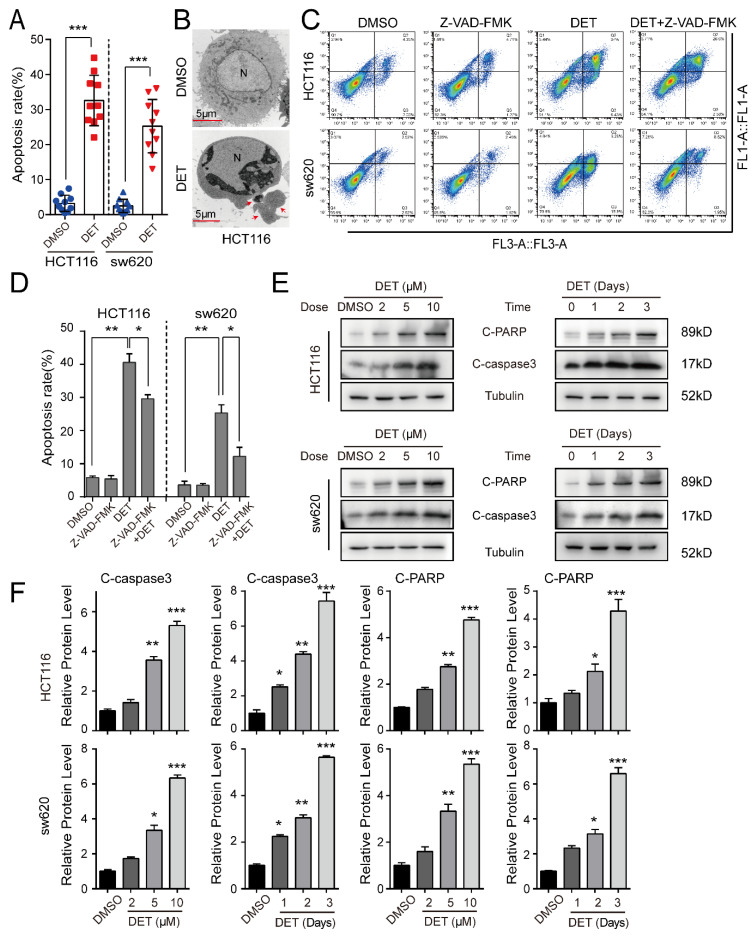
DET induces apoptosis in colon cancer cells. (**A**) The cell apoptosis level of DET-treated HCT116 and sw620 cells as detected by TUNAL assay. (**B**) Scanning electron microscope images of sw620 cells treated with DET (5 µM) or DMSO for 48 h. Arrows highlight the apoptosis bodies. Scale bar = 5 µm. (**C**,**D**) The apoptosis was determined by Annexin V-FITC/PI staining and flow cytometry. (**E**) Western blot assay was performed to assess apoptosis-related protein levels in the indicated concentrations (0, 2, 5, 10 µM) and indicated times (0, 24, 48, 72 h). (**F**) Protein levels are estimated based on the grayscale value of protein bands and normalized with the grayscale value of Tubulin bands. All data are shown as the mean ± SD. A two-tailed unpaired Student’s *t*-test was executed. * *p* < 0.05, ** *p* < 0.01, *** *p* < 0.001, versus control.

Apoptosis is a key factor for inhibiting cell proliferation. The TUNAL assay elucidated significantly higher apoptosis levels of HCT116 and sw620 cells treated with DET as compared to the control group (Figure 3A). Meanwhile, the transmission electron microscope (Figure 3B) showed the apparent apoptotic bodies in colon cancer cells treated with DET. Next, the supernatant and adherent HCT116 and sw620 cells treated with DET were collected after 48 h. The cells were stained with an AnnexinV-APC kit. As shown in Figure 3C,D, DET-treated HCT116 and sw620 cells claimed a certain degree of early and late apoptosis, but there was no obvious apoptosis in the control group. At the same time, we found that the apoptosis level of HCT116 and sw620 cells treated with combined DET and Z-VAD-FMK (an inhibitor of apoptosis) was lower than that of the DET group alone. The results of Western blot documented a dose-dependent and time-dependent increase in the expression levels of marker proteins of apoptosis, C-caspase3 and C-PARP, upon DET treatment (Figure 3E,F).

### 2.4. DET Induces Apoptosis by Inhibiting Bcl2 in Colon Cancer Cells

**Figure 4:**
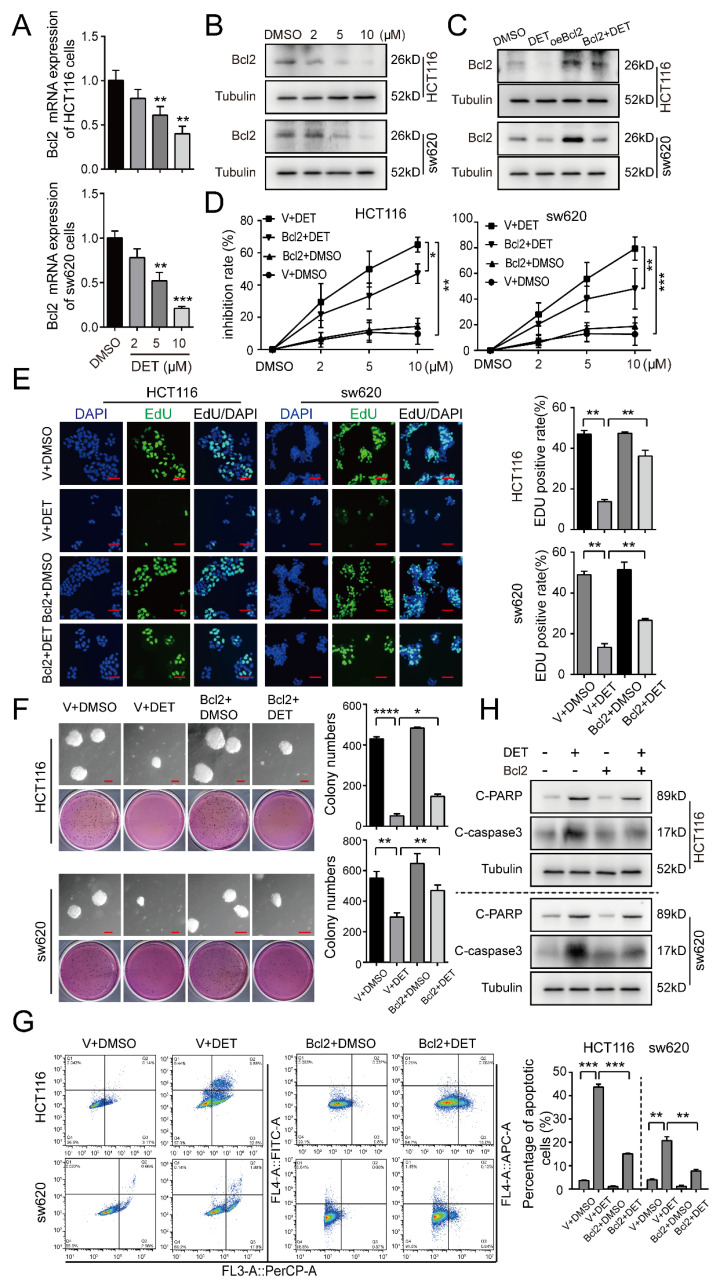
DET induces apoptosis by inhibiting Bcl2 in colon cancer cells. (**A**) qRT-PCR assay to detect the expression of Bcl2. (**B**) The expression of Bcl2 in HCT116 and sw620 cells treated with different concentrations of DET for 48 h. (**C**) The expression of Bcl2 in 5 µM DET-treated HCT116 and sw620 cells with Bcl2-overexpressed or empty vector. (**D**) Graphic representation of results from MTT assays to determine cell inhibition rate of Bcl2-overexpressed HCT116 and sw620 cells treated with different concentrations of DET for 72 h. (**E**) EdU-positive cells in Bcl2-overexpressed HCT116 and sw620 cells after treatment with 5 µM DET. Scale bar = 20 µm. Quantification of EdU-positive HCT116 and sw620 cells also shown in the panel. (**F**) Soft agar assay was performed to assess colony formation ability of Bcl2-overexpressed HCT116 and sw620 cells. Scale bar = 100 µm. Colony numbers in the panel were quantified. (**G**) Bcl2-overexpressed HCT116 and sw620 cells were treated with DMSO and 5 µM DET for 48 h and apoptosis was determined by Annexin V-FITC/PI staining and flow cytometry. (**H**) The expression of apoptosis proteins was detected in Bcl2-overexpressed HCT116 and sw620 cells after treatment with DET for 48 h. All data are expressed as the mean ± SD. A two-tailed unpaired Student’s *t*-test was carried out. * *p* < 0.05, ** *p* < 0.01, *** *p* < 0.001, **** *p* < 0.0001, vs. control.

Treating HCT116 and sw620 cells with a concentration gradient of DET, the mRNA protein levels of the anti-apoptotic protein Bcl2 were found to be reduced significantly and stably (Figure 4A,B). Therefore, we presumed Bcl2 to be a pivotal factor in DET-mediated apoptosis. Next, we employed lentiviruses expressing Bcl2 to stably infect HCT116 and sw620 cells. The Western blot assay confirmed Bcl2 overexpression after viral infection, and DMSO and empty vector treatments were used as the controls (Figure 4C). The MTT assay showed that the cell inhibitory rate of the Bcl2 overexpression group was higher than that in the control group, while it was lower than the DET group in HCT116 and sw620 cells (Figure 4D). Further, we verified that DET and HA14–1 (Bcl2-specific inhibitor) exhibited synergistic curative effects on colon cancer cells by Jin’s formula (q value ≥ 1.15) (Figure S1A). Likewise, the results of EdU, as illustrated in Figure 4E, revealed that the EdU-positive signals of the Bcl2 overexpression group were greater than in the DET group alone in HCT116 and sw620 cells. This indicates that Bcl2 overexpression might partially restore the DNA replication inhibited by DET. The soft agar assay also substantiated a similar outcome wherein the clone spots in the Bcl2-overexpressed HCT116 and sw620 cells treated with DET were smaller and fewer than those in the control group, but larger and more numerous in comparison to the colon cancer cells treated with DET (Figure 4F).

Furthermore, the results of apoptosis detected by flow cytometry also verified that the overexpression of Bcl2 could partly prevent the apoptosis of HCT116 and sw620 cells induced by DET (Figure 4G). Consistent with the previous conclusions, Western blot also revealed that, compared with the DET group, the expression of C-caspase3 and C-PARP in the Bcl2-overexpressed HCT116 and sw620 cells was significantly lower (Figure 4H). Moreover, the combined treatment results demonstrated that the combined DET and HA14–1 group had higher protein expression levels than the group with or without HA14–1 in HCT116 and sw620 cells (Figure S1B). The in vivo experimental results from Figure S1C,D also indicate that the tumor weight and volume of mice in the DET group and the combined treatment group were lower compared with the control group, while the combined treatment group was lower than the DET group and there were significant differences between the two groups. These findings were further substantiated by the results of IHC staining, which revealed diminished Ki-67 proliferation signals in the combined group compared to those in the DET group. However, there was no significant difference between the control group and the HA14–1 group (Figure S1E,F). This implies that the combination of DET and HA14–1 can enhance the apoptosis of colon cancer induced by DET. These results indicate that the DET-mediated inhibition of Bcl2 expression induced the apoptosis of colon cells.

### 2.5. miR-205 Is Directly Targeted by Bcl2 and Induces the Apoptosis of Colon Cancer Cells

**Figure 5:**
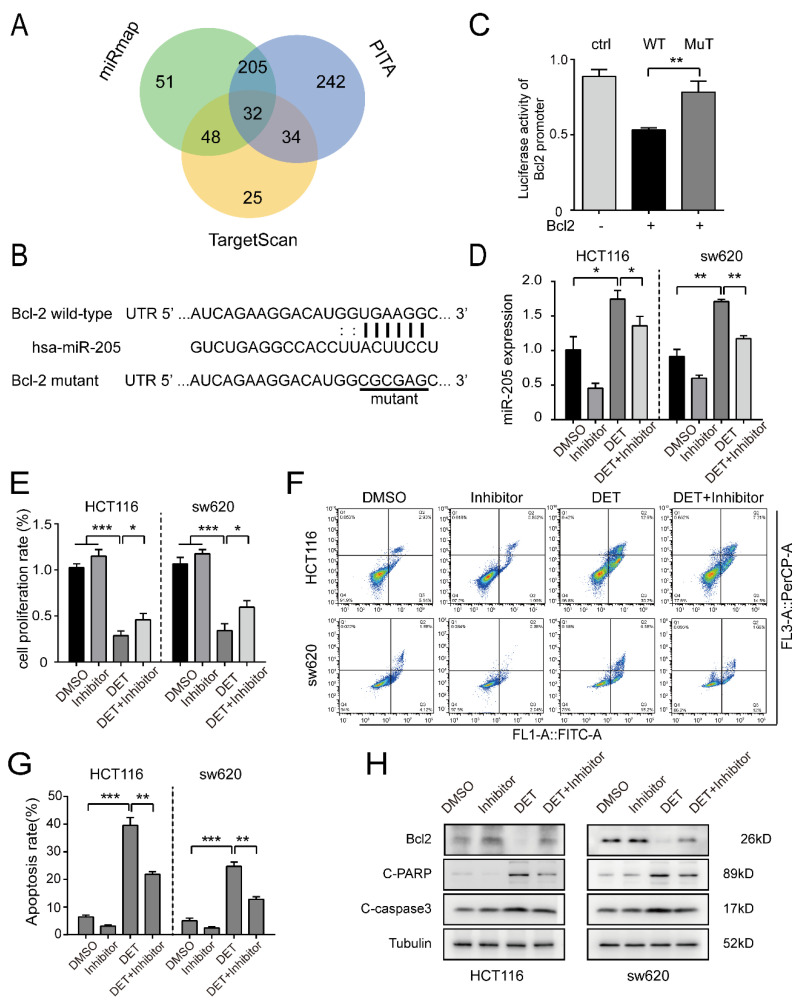
miR-205 is directly targeted by Bcl2 and induces the apoptosis of colon cancer cells. (**A**) Venn diagram showing the potential miR-205 targets predicted by databases. (**B**). The putative binding site of miR-205 and Bcl2 is shown. A schematic of the construction of wild-type or mutant pGL3-Bcl2 3′-UTR vectors is indicated. (**C**) The 293FT cells were co-transfected with miR-205 and wild-type or mutant Bcl2 3′-UTR fused to the Renilla luciferase vector. The relative firefly luciferase activities were determined. (**D**) The expression of miR-205 after DET treatment or DET and the miR-205 inhibitor treatment for 48 h. (**E**) Cell proliferation of HCT116 and sw620 cells was promoted by miR-205 inhibitor. (**F**,**G**) Flow cytometry assay showing that miR-205 inhibitor significantly reversed the apoptosis of cell metastasis in HCT116 and sw620 cells. (**H**) Western blot assays were used to detect the expression of proteins after treatment with the miR-205 inhibitor. All data were used as mean ± S.D., n = 3, significant difference was examined by Student’s *t*-test. * *p* < 0.05, ** *p* < 0.01, *** *p* < 0.001, versus control. Note: Inhibitor in this figure refers to the inhibitor of miR-205.

Bioinformatics analysis in three databases revealed 32 genes to be potential targets of miR-205 (Figure 5A). A putative binding site for miR-205 was identified in the 3′-UTR of Bcl2 (Figure 5B). We constructed reporter plasmid vectors containing the wild type or mutant seed sequence in the 3′-UTR fragment of Bcl2 and found that the luciferase activity of the wild-type reporter was significantly reduced by miR-205 transfection in 293FT cells, while it remained unchanged in the mutant reporters, suggesting that miR-205 acts directly on downstream target Bcl2 (Figure 5C). From the real-time quantitative fluorescence PCR, shown in Figure 5D, it is quite prominent that the expression of miR-205 was downregulated after inhibitor treatment. Furthermore, compared with inhibitor treatment alone, the expression of miR-205 in the cells treated with inhibitor and DET was higher. This indicated that DET might induce miR-205 expression. Moreover, the results of the MTT assay, as detailed in Figure 5E, confirmed that the downregulation of miR-205 could partly rescue the survival rate of colon cancer cells. Further, we collected the floating and adherent cells of the experimental group and the control group by flow cytometry and found that the apoptosis in the combined miR-205 inhibitor and DET group was significantly lower as compared to that in the DET group alone (Figure 5F,G). Western blot detection (Figure 5H) also confirmed that the downregulation of miR-205 promotes the expression of Bcl2 and prevents the apoptosis of HCT116 and sw620 cells induced by DET. Thus, DET could induce the apoptosis of colon cancer cells via the miR-205-Bcl2 axis.

### 2.6. DET Enhances Chemosensitivity to 5-Fluorouracil (5FU) in Colon Cancer Cells

**Figure 6:**
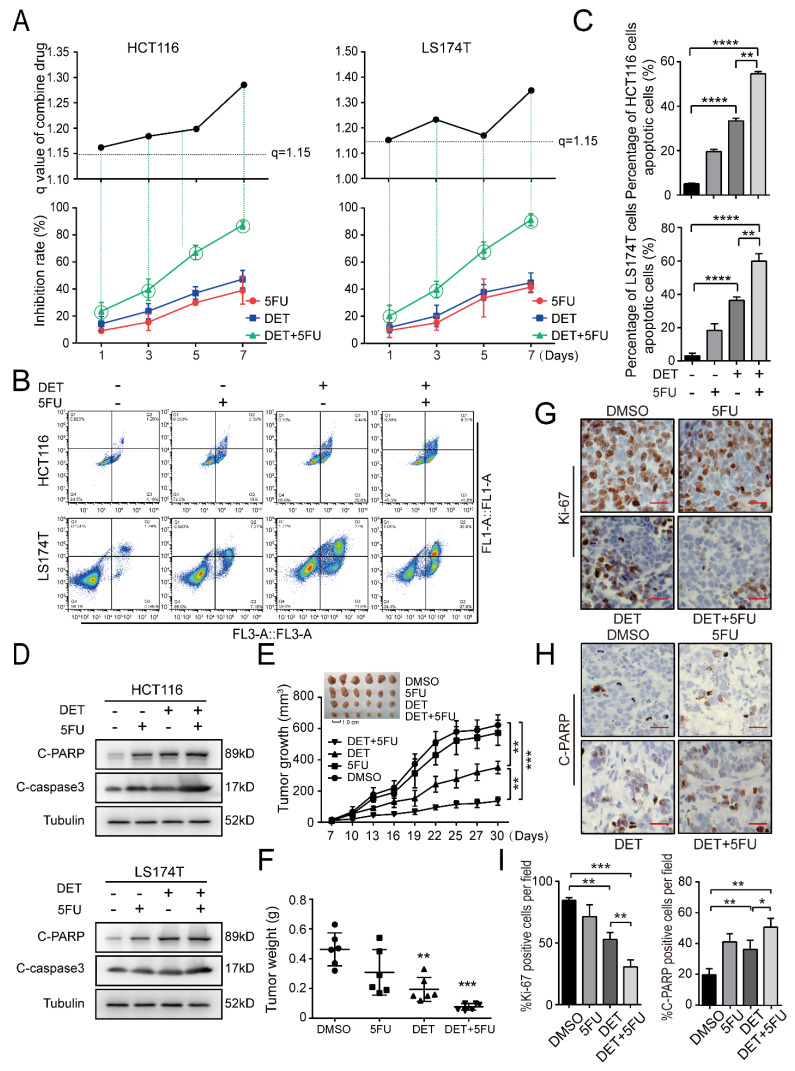
DET enhances chemosensitivity to 5-Fluorouracil (5FU). (**A**) A panel of colon cell lines was treated with 5 µM DET, with or without 5FU (5 µM), for 1, 3, 5, 7 d. (**B**) HCT116 and LS174T cells were treated with 5 µM DET, with or without 5FU (5 µM), and the apoptosis was detected with flow cytometry. (**C**) Quantification results of apoptosis rate of HCT116 and LS174T cells. (**D**) HCT116 and LS174T cells were treated with DET, with or without 5FU, and then the expression levels of apoptosis proteins were explored by Western blot assay. (**E**) The volume changes of xenograft tumors treated with DET, with or without 5FU. (**F**) Tumor weight of the xenograft tumors in each group. (**G**,**H**) The nuclear tumor staining intensity of Ki-67 and C-PARP was evaluated using immunohistochemistry. (**I**) Positive expression rates of the Ki-67 and C-PARP in (**G**,**H**). Scale bar = 20 µm. All data were expressed as mean ± S.D., n = 3, significant difference was verified by Student’s *t*-test. * *p* < 0.05, ** *p* < 0.01, *** *p* < 0.001, **** *p* < 0.0001, versus control.

It is well known that 5-Fluorouracil (5FU) is a pyrimidine analog that interferes with thymidylate synthesis and has clear activity against colon tumors in clinical practice. However, the drug resistance of 5FU reduces its therapeutic efficacy [28,29]. The synergy between 5FU and DET was noted over a broad range of time. The synergistic effect of DET and 5FU was noted in a wide range of time, indicated by q values of ≥1.15, adopting Jin’s formula. It was observed that treatment with 5FU and DET synergistically enhanced DET-mediated cell growth suppression (Figure 6A). Meanwhile, apoptosis detected by flow cytometry also showed that the apoptotic level in the combined group was greater than that in the 5FU group or DET group alone in HCT116 and LS174T cells (Figure 6B,C). The results of Western blot further substantiated that the combined 5FU and DET treatment group had higher C-caspase3 and C-PARP protein expression than those treated with DET or 5FU alone in HCT116 and LS174T cells (Figure 6D). To extend these in vitro findings to in vivo tumor growth, the combination of DET and 5FU in immunodeficient mice injected with HCT116 cells was investigated. A month later, tumors obtained from mice treated with the combination of DET and 5FU were found to be significantly smaller than tumors from mice treated with DET or 5FU alone (Figure 6E,F). Additionally, the combination, with a stronger effect of promoting apoptosis, had a more significant impact than DET alone on tumor growth (Figure 6G,H). These findings suggest that DET could enhance the chemosensitivity to 5FU of colon cancer cells and provide a theoretical basis for the more extensive clinical treatment of colon cancer.

## 3. Discussion

The ethanol extracts of *E. scaber* significantly inhibited the growth of cancer cells and induced apoptosis [30]. One of the main bioactive compounds isolated from *E. scaber* is deoxyelephantopin, which inhibits the development of human cancer cells [31,32]. The monomer has been found to impede the growth of breast cancer [10], lung cancer [33], lymphoid cancer [34], and nasopharyngeal carcinoma [32], and induce apoptosis of osteosarcoma cells [35] and hepatoma cells [9]. This study further consolidates the prominent anti-cancer activity of DET both in vivo and in vitro. At the same time, we also found that DET blocked the cell cycle of colon cancer in the G2/M phase, which may be caused by DET-induced DNA damage. Several studies have identified miR-205 as a radiosensitizing miRNA that directly or indirectly inhibits DNA damage repair [36,37,38]. These prompted us to consider whether the DET-induced upregulation of miR-205 in this study promotes colon cancer cell apoptosis through some functional targets of miR-205 in response to DET-induced DNA damage, which remains to be further explored.

We found that DET mediated the steady and dramatic inhibition of Bcl2 expression at the mRNA level and protein level in the process of elucidating the apoptosis of colon cancer cells induced by DET. During tumorigenesis and cancer progression, cancer cells rely on the dysregulation of the BCL2 protein family and tend to survive [39,40]. For example, somatic copy number amplification of both the MCL1 and BCLxL genes in human cancers is an instance of such imbalance [41]. We ascertained that Bcl2 overexpression could support the survival of colon cancer cells upon DET treatment. In line with these findings, our data reveal that the combination of Bcl2 inhibitor HA14–1 and DET was more effective than DET alone in inhibiting the growth of colon cancer cells both in vitro and in vivo. These data explain the mechanisms of the DET-induced apoptosis of colon cancer and reveal the vital role of Bcl2 in this process.

Many studies have substantiated that microRNA is involved in the regulation of biological growth and development and plays a primary role in the occurrence and development of tumors [42,43,44]. Thus, we predicted that DET could downregulate Bcl2 expression at the transcriptional level. Our work suggested that miR-205, which directly interacts with Bcl2, is upregulated in colon cancer cells upon DET treatment. Additionally, the inhibitor of miR-205 could recover Bcl2 expression and reduce DET-induced apoptosis in human colon cancer cells.

Research on the structure–activity relationship of plant sesquiterpene lactones has shown that the presence of an alkylating center (α-methylene-γ-lactone, α-methylene-δ-lactone, conjugated cyclopentenone, or conjugated side chain ester) is essential for their anti-cancer and immunomodulatory activity [45,46,47]. We found that the compound structure of DET contains multiple Michael acceptor structures through medicinal chemical structure analysis, which may be important pharmacodynamic functional groups. However, whether it exerts its effect through the covalent binding of Bcl2 or other protein cysteines needs further investigation.

The discovery of 5FU is a blessing for many cancer patients, but the resistance of the tumor limits the therapeutic effect [28,40,48]. The most exciting finding of our current study was that the combination of DET and 5FU had a better tumor-inhibitory effect on colon cancer. Collectively, DET can significantly enhance the chemosensitivity of the chemotherapeutic medicine 5FU to colon cancer cells and has an ideal tumor-inhibitory effect.

## 4. Materials and Methods

### 4.1. Preparation of DET

DET was purchased from Chengdu Herbpurify Co., Ltd., Chengdu, China. The chemical purity of DET was over 98% as judged by NMR spectrometry.

### 4.2. Lentiviral Constructs and Infection

The full-length coding sequence of overexpressed Bcl2 was ligated into the pCDH-CMV-MCS-EF1-puro vector by Wuhan GeneCreate Biological Engineering Co., Ltd. After this, lentivirus constructs, including pCDH-CMV-MCS-EF1-puro-Bcl2, empty vector, and packaging plasmid (pLP1,pLP2,PLP/VSVG), were transfected into 293FT cells by ViaFect transfection reagent (Promega, Madison, WI, USA) [49]. The virus supernatant was collected after two days. Thereafter, HCT116 and sw620 cells were infected with the virus-containing supernatant. After infection, the cells were cultured in the presence of 2 µg/mL puromycin (Life Technologies; Thermo Fisher Scientific, Inc, Waltham, MA, USA) twice. Finally, the drug-resistant cells were pooled.

### 4.3. RNA Sequencing and qRT-PCR

HCT116 and sw620 cells (American Type Culture Collection, ATCC).were treated with DMSO or DET and incubated in a 5% CO_2_ incubator at 37 °C. After two days, the cells were harvested, and the total RNA in HCT116 and sw620 cells was extracted with Trizol reagent, following the manufacturer’s protocol [50]. After this, the extracted RNA was used for reverse transcription and qRT-PCR according to the operation and instructions of the reverse transcription and quantitative kit (TakaraBio Inc., Kusatsu, Japan).

### 4.4. Cell Proliferation Analysis

The cell growth curve was analyzed by MTT. To determine the viability of the cells, 800 cells were cultured in a 96-well plate for 1, 3, 5, and 7 days and the cells were estimated by the MTT method at the fixed time point [51]. All the experiments were repeated three times independently.

### 4.5. Detection of Phosphatidylserine Externalization by Annexin V and PI Staining

HCT116 and sw620 cells were seeded in 100 mm^2^ culture dishes and incubated in the 5% CO_2_ incubator at 37 °C for 48 h. The cells were then treated with 5 mmol/L DET, while the negative control was treated with vehicle DMSO. After 48 h of treatment, both adherent and suspension cells were harvested and washed once with PBS and once with 1× Annexin V binding buffer. After this, cells were resuspended in 1× Annexin V binding buffer. Subsequently, the treated cells were stained with Annexin V-FITC and PI (50 µg/mL). The cells were then vortexed and incubated in the dark at room temperature for 20~30 min. The cells were then analyzed by flow cytometry using quadrant statistics for apoptotic cell populations.

### 4.6. Western Blot Assay

The treated cells were collected and certain amounts of protease inhibitor (Roche), phosphatase inhibitor (Sigma Aldrich, St. Louis, MO, USA), and RIPA lysate (Beyotime, China) were added based on the amount of the cells. The cells were then cracked in ice for 1 h and centrifuged. The total content of protein was elucidated by using the Bradford assay, and 50 mg of protein was subjected to 12% SDS-PAGE to separate the protein. After electrophoresis, the proteins were transferred onto a PVDF membrane (Millipore, Kenilworth, NJ, USA), followed by blocking using skim milk/BSA for 2 h, and then incubated with primary antibodies at 4 °C overnight. Subsequently, the membrane was incubated with the corresponding secondary antibody for 2 h at room temperature. For detection, the membrane was incubated with the help of an enhanced chemiluminescence (ECL) detection kit, and detection analysis system (Clinx Science, Shanghai, China) were used to visualize and capture proteins. [52].

### 4.7. EdU Staining

The colorectal cancer cells in 24-well plates were treated with DET. When there was a significant difference in the cell phenomenon between the control group and the experimental group, 10 µL of 10 mmol/L EdU (5-Ethynyl-2′-deoxyuridine, from Invitrogen, Carlsbad, CA, USA) was added to each well. After this, the plates were kept in an incubator for 30 min and then fixed with 4% PFA for 15 min. After punching holes with 0.5% TritonX-100 at room temperature for 20 min, 0.5 mL of Click-iT reaction cocktail was added to each well and incubated away from light for 30 min at room temperature. Next, the nuclei were stained with DAPI. EdU-positive cells in random fields were counted.

### 4.8. Soft Agar

The ability of colony formation was conducted on HCT116 and sw620 cells by the soft agar assay. First, 1.5 mL 2 × DMEM medium containing 0.6% agarose was added to six-well plates [53]. Next, 1000 cells in the logarithmic phase mixed with a medium containing 0.3% agar and different concentrations of DET were added to the bottom glue [54]. After three weeks of culture at 37 °C in CO_2_ incubators, colonies were captured by microscopy and stained with MTT, and scanned with a scanner. Each sample in this experiment was assessed in triplicate.

### 4.9. Luciferase Reporter Assay

For the luciferase reporter assay, 293FT cells were co-transfected with 200 nM miR-205 mimic or NC (RiboBio Co., Ltd., Guangzhou, China) and 650 ng of pGL3-Basic-Bcl2-3′-UTR-WT, pGL3-Basic-Bcl2-3′-UTR-MUT. Cells were collected 48 h after transfection and analyzed with the Dual-Luciferase Reporter Assay System (Yeasen Biotechnology Co., Ltd., Shanghai, China). Moreover, 293FT cells were co-transfected with the pGL3-Basic vector and NC was used as a control.

### 4.10. Animal Studies

All animal experiments were performed under the Guidelines of the Institute for Laboratory Animal Research, Southwest University (Chongqing, China). Twelve adult immunodeficient mice were injected with HCT116 cells at the left and right armpits on day 1. Tumors became visible after a week. Next, drugs or saline were injected intraperitoneally once every two days for half a month. DET (30 mg/kg) and free HA14–1 (400 nmol) were dissolved in DMSO before injection. Tumor volume (V) was measured with calipers before each injection and calculated by the formula V = (LXW^2^)/2, where L is the length and W is the width of the tumor [55].

### 4.11. Animal Ethics

Twelve adult immunodeficient mice (Hunan Slike Jingda Laboratory Animal Co., Ltd., Changsha, China, animal qualification number: SCXK-2019-0004), body weight (25 ± 20) g, were used. The animal experiments in this study were carried out in the Institute for Laboratory Animal Research, Southwest University, and approved by the Ethics Committee of Experimental Animals of Southwest University (approval number IACUC-20190320-02).

### 4.12. Statistics Analysis

GraphPad was employed for statistical analysis. All experiments were confirmed using at least three independent experiments. All the results in this study are represented as the mean ± standard deviation (SD). A significant difference was observed in Student’s unpaired *t*-test. *p* < 0.05 was considered to indicate a statistically significant result.

## 5. Conclusions

This study confirms the anti-human colon cancer activity of DET in vivo and in vitro and elucidates that DET can induce apoptosis of colon cancer by the miR-205-Bcl2 axis, as well as enhance the chemosensitivity of 5FU to colon cancer, which has a bright prospect for the treatment of colon cancer patients.

## 6. Patent

Not applied.

## Data Availability

The data presented in this study are available on request from the corresponding author.

## Supplementary Materials

The following supporting information can be downloaded at: https://www.mdpi.com/article/10.3390/ijms23095051/s1.

## Abbreviations

DET	Deoxyelephantopin
5FU	5-Fluorouracil
Bcl2	B-cell lymphoma-2
CC	Colon Cancer
IHC	Immunohistochemistry
MTT	3-(4,5-Dimethylthiazol-2-yl)-2,5-diphenyltetrazolium bromide
DMSO	Dimethyl sulfoxide
