# Supplementary material for: Deoxyelephantopin Induces Apoptosis and Enhances Chemosensitivity of Colon Cancer via miR-205/Bcl2 Axis
Source: Int J Mol Sci. 2022 May 2;23(9):5051. doi: 10.3390/ijms23095051 (PMC9099879; doi:10.3390/ijms23095051)
Supplement: Supplementary file 1 [file ijms-23-05051-s001.zip › ijms-1677802-supplementary.pdf]

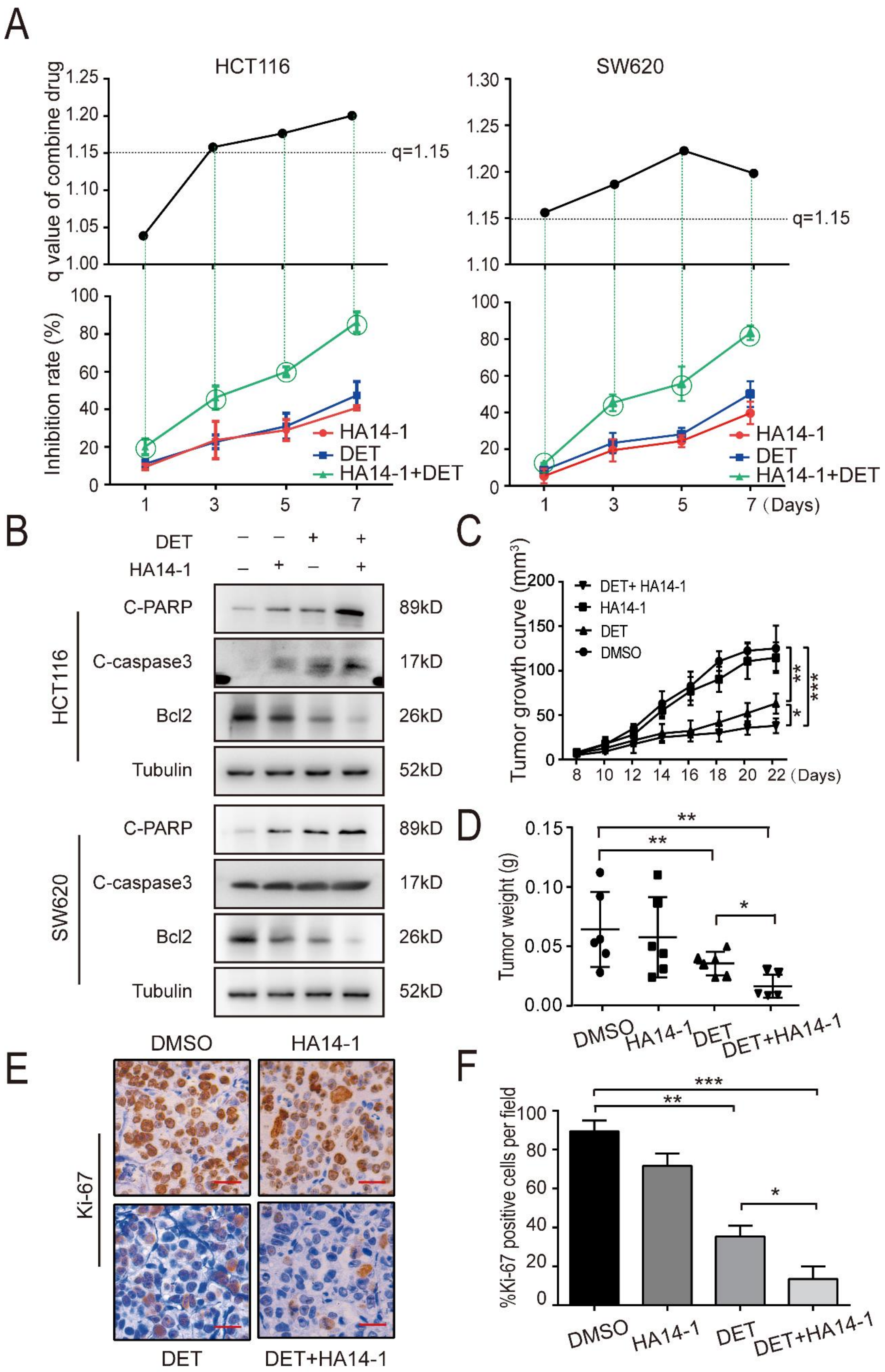

Figure S1. A combination of DET and HA14–1 induces apoptosis and inhibits the growth of colon

cancer cells

(A) HCT116 and SW620 cells were treated with 5 μM DET, with or without HA14–1, and then the expression levels of apoptosis proteins were detected using Western Blot assay. (B) Xenograft tumors were removed from immunodeficient mice treated with DET, with or without HA14–1 for two weeks. (C) The volume changes of xenograft tumors treated with DET, with or without HA14–1. (D) Tumors weight of the xenograft tumors in each group. (E and F) The nuclear tumor staining intensity of Ki-67 was evaluated using immunohistochemistry. Scale bar = 20 μm. All data were represented as mean ±S.D., n = 3, significant difference was evaluated by student's t-test. *p<0.05, **p<0.01, ***p<0.001, versus control.
